# Cost-effectiveness of active transport for primary school children - Walking School Bus program

**DOI:** 10.1186/1479-5868-6-63

**Published:** 2009-09-14

**Authors:** Marjory Moodie, Michelle Haby, Leah Galvin, Boyd Swinburn, Robert Carter

**Affiliations:** 1 Deakin Health Economics, Public Health Research Evaluation and Policy Cluster, Deakin University, 221 Burwood Highway, Burwood, Victoria 3125, Australia; 2 Public Health Branch, Department of Human Services, 50 Lonsdale Street, Melbourne, Victoria 3000, Australia; 3 School of Exercise and Nutrition Sciences, Deakin University, 221 Burwood Highway, Burwood, Victoria 3125, Australia; 4 WHO Collaborating Centre for Obesity Prevention, Deakin University, 221 Burwood Highway, Burwood, Victoria 3125, Australia

## Abstract

**Background:**

To assess from a societal perspective the incremental cost-effectiveness of the Walking School Bus (WSB) program for Australian primary school children as an obesity prevention measure. The intervention was modelled as part of the ACE-Obesity study, which evaluated, using consistent methods, thirteen interventions targeting unhealthy weight gain in Australian children and adolescents.

**Methods:**

A logic pathway was used to model the effects on body mass index [BMI] and disability-adjusted life years [DALYs] of the Victorian WSB program if applied throughout Australia. Cost offsets and DALY benefits were modelled until the eligible cohort reached 100 years of age or death. The reference year was 2001. Second stage filter criteria ('equity', 'strength of evidence', 'acceptability', feasibility', sustainability' and 'side-effects') were assessed to incorporate additional factors that impact on resource allocation decisions.

**Results:**

The modelled intervention reached 7,840 children aged 5 to 7 years and cost $AUD22.8M ($16.6M; $30.9M). This resulted in an incremental saving of 30 DALYs (7:104) and a net cost per DALY saved of $AUD0.76M ($0.23M; $3.32M). The evidence base was judged as 'weak' as there are no data available documenting the increase in the number of children walking due to the intervention. The high costs of the current approach may limit sustainability.

**Conclusion:**

Under current modelling assumptions, the WSB program is not an effective or cost-effective measure to reduce childhood obesity. The attribution of some costs to non-obesity objectives (reduced traffic congestion and air pollution etc.) is justified to emphasise the other possible benefits. The program's cost-effectiveness would be improved by more comprehensive implementation within current infrastructure arrangements. The importance of active transport to school suggests that improvements in WSB or its variants need to be developed and fully evaluated.

## Background

Active transport, to and from school, offers an opportunity for school aged children to increase their levels of daily physical activity. This can lead to physical, mental health, safety and environmental benefits for both the community and participating individuals [1]. While the majority of Australian primary school children in urban environments live within walking and cycling distance to school [2], there are a number of real and perceived barriers which contribute to the continued decline in active transport rates. These include perceived danger to children from strangers, inadequate safe crossing points and the presence of heavy traffic [3]. The increasing participation of women in the workforce adds time pressure to family living [4] and also potentially contributes to declining active transport rates.

In countries such as The Netherlands and Germany, where there is a culture of active transport and compulsory education programs for children around active transport and traffic safety, participation rates are much higher and obesity, diabetes and hypertension rates (in adults) are much lower than the USA or Australia [5]. Government strategies in The Netherlands and Germany to increase active transport and improve safety have seen fatalities decline markedly between 1975 and 2001 and urban environments changed to improve conditions for active transport participants [5].

As an active transport to school option, the Walking School Bus (WSB) program offers a potentially important strategy to reduce overweight and obesity in children. The purpose of this paper is to assess, within the context of the Assessing Cost-Effectiveness of Obesity (ACE-Obesity) project [6], the potential cost-effectiveness of the WSB program as an obesity prevention measure for primary school children. The program was not intended as a dedicated obesity prevention measure, and this study acknowledges its multiple benefits. Whilst not endeavouring to quantify them, we investigated the impact of attributing a share of the program costs to non-health objectives.

## Methods

### Overview

A cost-effectiveness evaluation was undertaken, and the incremental cost-effectiveness ratio (ICER) calculated as the cost ($AUD) per Body Mass Index (BMI) unit saved and disability-adjusted life year (DALY) saved. The intervention time horizon was one year, which reflects how the program would be applied in real life. It was assumed to be in steady state (i.e. it is implemented and working at its full effectiveness potential and trained personnel or infrastructure are available). The time horizon for measuring the associated health care cost-offsets and DALY benefits was rest of life or 100 years. All costs and benefits were discounted at 3% in accordance with the recommendations of the US Consensus Panel on Cost-Effectiveness [7]. The reference year was 2001. In addition to the technical results, the intervention was assessed against a series of second stage filter criteria ('equity', 'strength of evidence', 'acceptability', 'feasibility', 'sustainability' and 'side-effects'). This analysis involves the assessment of issues that either influence the degree of confidence that can be placed in the cost-effectiveness ratio, or broader issues that need to be taken into account in the decision-making about resource allocation. The economic evaluation methods are detailed in a separate paper (submitted to *BMC Public Health 25 March 2009*) and in [8].

### The Intervention

The proposed intervention was based on the WSB program as operated under the auspices of VicHealth in Victoria, Australia. The program aims to increase the number of children of primary school age walking to school [9]. Children are accompanied by 2 volunteer adult "conductors" (at a ratio of 1 adult to 8 children) and travel along a set route through a neighbourhood picking up children along the way at designated stops and delivering them to school. Conductors complete an induction program, are registered and insured under their local government's volunteer policy. All volunteers must complete a satisfactory police check [9].

### Current Practice

The comparator for this intervention was 'current practice', defined as 'do nothing' as there was no organised program was in place. Before implementation of the WSB in Victoria, approximately 27% of Preparatory to Grade 2 children (ages 5-7 years) and 24% of Grade 3 to 6 children (8-11 years) walked to school every day [1].

### Assessment of Benefit

The first stage of benefit assessment involved estimating the health gain attributable to the intervention using the DALY. This required calculation of the increase in physical activity due to the intervention, conversion to BMI change as children and then conversion to DALYs and cost-offsets over their lifetime [6]. There are no reported trials of the impact of the WSB interventions on physical activity, weight or BMI. Therefore, a range of other available data was used to model the likely change in the BMI of individual participants of the WSB program who were new to active transport (Table 1). It was assumed that there was no effect of a change from car transport to active transport on physical activity at other times or energy intake levels [10-12].

**Table 1:** Modelling of reduction in BMI for a single 'average' individual new to active transport (Effects are averaged over one calendar year)

	**Prep to Grade 2**		**Comments**
	**Boys**	**Girls**	
Height (m)	1.20	1.19	Mean height for 5-7 year age group [19]
Weight (kg)	23.50	23.48	Mean weight for 5-7 year age group [19]
Body mass index BMI (kg/m2)	16.24	16.47	Mean BMI for 5-7 year age group [19]
Estimated total energy expenditure (MJ/day)	6.43	6.64	Total energy expenditure (MJ/day) = [.107 × weight (kg)] + [2.91 × height (metres)] + .417 [20]
Estimated total energy expenditure (kJ/day)	6433	6404	Conversion to kilojoules - multiply by 1000
Increased METS -- walking versus sitting	2.5	2.5	Metabolic equivalents for sitting = 1.0, walking 3.5. Therefore, additional energy expenditure of active transport to school = 2.5 METS [13]
Extra time spent on walking to and from school (min)	28.30	28.30	Mean travel time for Victorian children participating both in morning and afternoon WSB [15]
Energy expenditure increase from WSB participation(kJ per day)	116	116	Increase in individual energy expenditure from walking (kJ/school day) = weight (kg) × increased METs × time (hrs) × factor to convert kcal to kJ (4.2)
Average number of days of WSB participation to and from school per week	1	1	Based on Victorian WSB experience 2004 [15], where 1,675 enrolled children engaged in 3,049 sessions of active transport per week. This translates to 1.82 sessions per week/child, or about equivalent of walking to and from school one day per week.
Number of potential weeks of WSB participation per year	40	40	Number of weeks in the school year.
Total number of days of WSB participation per year	40	40	Number of WSB days per week × number of school weeks
Energy expenditure increase from WSB participation (kJ)	13	13	Total increase in individual energy expenditure × number of WSB days per year divided by 365
Relative increase in energy expenditure with WSB participation (%)	0.20	0.20	Average individual energy expenditure from WSB as % of estimated total energy expenditure per day: (13/6433) × 100 and (13/6404) ×100 for boys and girls respectively
Conversion factor	0.45	0.45	Factor for conversion of relative change in energy balance to relative change in weight [14,21]
Relative lower weight with WSB participation (%)	0.09	0.09	[1-(energy expenditure_1_/energy expenditure_2_)^0.45^]*100
Absolute lower weight with WSB participation (kg)	0.02	0.02	0.09% of original weight
New weight (kg)	23.48	23.46	Original mean weight minus decrease in weight as a result of WSB participation
New BMI (kg/m2)	16.23	16.45	New weight divided by square of height
Reduction in BMI (kg/m2)^a^	0.014	0.015	Original BMI minus new BMI for new participants

^a^These figures are point estimates, which do not take into account uncertainty around any of the input parameters. As a result, they are different to the BMI changes quoted in the results section.

BMI body mass index; METS metabolic equivalent units; WSB Walking School Bus; kJ kilojoules; kg kilograms; m^2 ^metres squared

The increased energy expenditure for a child who changed from car transport to WSB was calculated by subtracting the energy costs of walking (3.5 metabolic units [METS]) from the energy costs of sitting in a car (1.0 METS) [13]. The net 2.5 METS was multiplied by the assumed average weight of the target age children (kg) and the assumed time to walk to and from school to derive total increased energy expenditure (kJ/d) (Table 1). The validated method of Swinburn et al. was used to convert changes in energy balance to changes in weight [14].

Whilst VicHealth had data on the percentage of children walking to school pre-intervention and on current participation of children involved in the Victorian program, there were no data available about the consequent change in active transport rates or the number of participants new to active transport. As anecdotal evidence indicated that some participants previously walked to school with a parent, it was assumed that, of the children participating in the WSB intervention, 50% were new to active transport. However, an uncertainty range of 25-75% was placed around this parameter.

### Simulation of the intervention

#### Delivery model

VicHealth funds local municipalities to implement the WSB program. Local governments, in collaboration with the schools and various State authorities and community organisations, facilitate establishment of the walking routes, registration of volunteers and liability insurance for volunteers. Each local government must engage at least four local primary schools during the project. The schools are responsible for the recruitment of both volunteer conductors and children to the program, and for the day-to-day operation of the program. The funding is dependent on the meeting of minimum standards, including the provision of police checks, insurance and training for volunteers and the safety auditing of routes.

#### Recruitment

**Figure 1:**
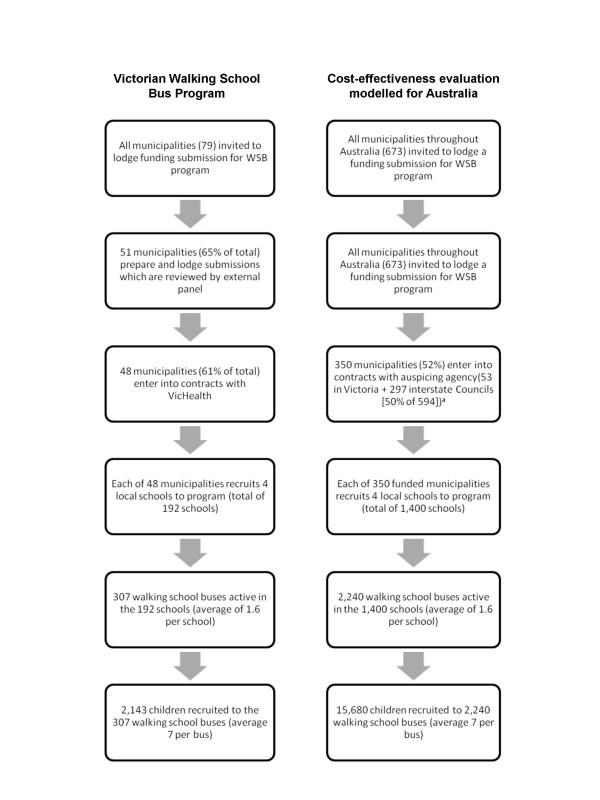
**Recruitment to the Walking School Bus intervention**. ^a ^Assumes lower percentage of municipalities involved than under the cost-efficacy trial

It was assumed that the delivery model reflected the intervention operating in 'steady state' in terms of recruitment, uptake and delivery. The current recruitment experience of the Victorian WSB Program [15], shown in the left hand column of Figure 1, was extrapolated to a national level (right hand column). However, the modelling assumed that fewer local governments in other states or territories would be involved in the program because a higher proportion of their population was rural or remote, and lower relevance of the program to their population. It was also assumed that enrolled children were confined to the first three years of primary school (Prep to Grade 2), as is typical of most enrolees. The number of volunteer conductors involved with a WSB ranges from one to five. For the purposes of training, four volunteers per bus were allowed for, giving a total of 8,960 volunteers.

The benefits at the individual level in terms of reduced BMI and DALYs saved were then applied to participating children new to active transport (50% × 15,680) from the Australian childhood population (age 5-7 years) [6].

### Assessment of costs

**Figure 2:**
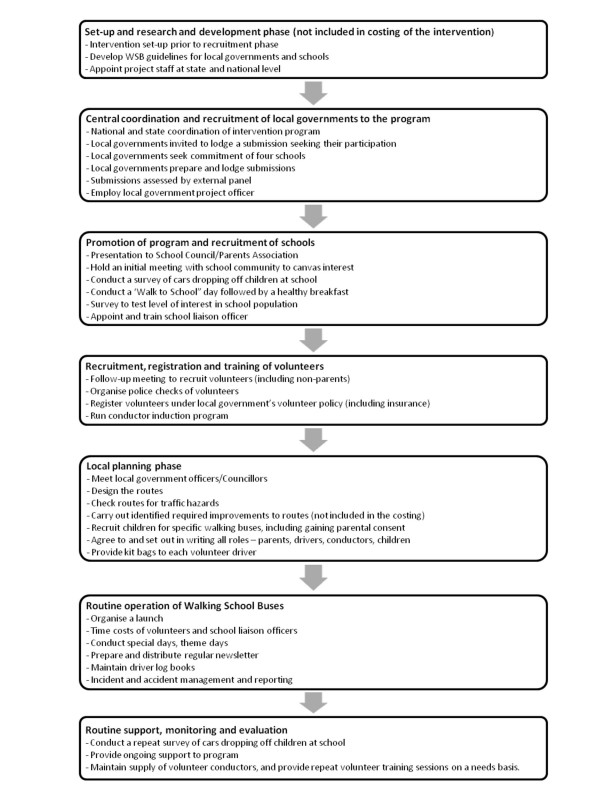
**Intervention pathway**.

Pathway analysis was used to identify the component activities of the intervention in order to ascertain the associated resource utilisation (Figure 2). The costs included, unit costs and their sources, and the assumptions employed are specified in Additional file 1. All costs were adjusted to real prices in the reference year using the relevant Consumer Price Index [16].

Given the societal perspective, costs to participants and their families, and all sectors involved in the delivery of the intervention were included (Figure 2, Additional file 1). Since the intervention was assumed to be operating in 'steady state', costs involved in set-up, research and development prior to the recruitment phase were not included. Other costs excluded were those associated with the implementation by local government of any improvements along proposed bus routes identified as necessary prior to commencement of a WSB and the costs of monitoring and evaluation above more than a routine level.

### Uncertainty analysis

Uncertainty analysis was used to address issues of uncertainty in the results due to sampling error (e.g. in the WSB 'snapshots') and the need to make assumptions due to the lack of evidence surrounding some parameters. The key parameters included in the uncertainty analysis are listed in Table 2. Simulation-modelling was used to facilitate the presentation of an uncertainty interval (median and 2.5 and 97.5 percentiles) around the health benefits, costs and ICERs. The @RISK software was used to conduct Monte Carlo simulations which allow multiple (4,000 in this instance) recalculations of a spreadsheet, each time choosing a value from the specified distribution for each input variable. The ranges presented can be interpreted as the range within which the true value lies with 95% certainty. In addition, the @RISK analysis allows identification of the model parameters that contribute most to the uncertainty in the results.

**Table 2:** Uncertainty analysis

**Parameters**	**Values**	**Uncertainty distribution**	**Sources and assumptions**
Height, weight of participants	Mean, SE^a^	Normal^b^	[19]
No. local governments making submissions	296, 370, 444^c^	Triangular^d^	Estimate
% non-Victorian local governments participating	0.4, 0.5, 0.6^3^	Triangular^d^	Estimate based on VicHealth figures for Victoria
No. participating schools per local government	4, 1^b^	Normal^b^	VicHealth
Persons attending recruitment evening per school	10, 15, 20^c^	Triangular^d^	Estimate
Volunteers recruited per school	7,3^b^	Normal^b^	Estimate
WSB per school	1, 1.6, 3^c^	Triangular^d^	Min, max -- estimates; most likely -- Vic Health
Volunteers trained per WSB	2, 4, 6^c^	Triangular^d^	Min, max -- estimates; most likely -- VicHealth guidelines
Average number of children per WSB	3, 7, 12^c^	Triangular^d^	Estimate
Extra minutes spent on walking	6, 28.3, 84^c^	Triangular^d^	[15]
No. of days of WSB per week per child	0.5, 1, 5^c^	Triangular^d^	Mean [15]
No. weeks of intervention effect per year	35, 40^e^	Uniform^f^	Estimate
Increased METS from walking	1.5, 2.5, 3^c^	Triangular^d^	[13]
Factor to convert % change in energy balance to % change in body weight	0.38, 0.45, 0.51^c^	Triangular^d^	[14]
% children enrolled in WSB not walking previously	25%, 50%, 75%^c^	Triangular^d^	Estimate
Local government officer time (days per week)	2, 3, 4^c^	Triangular^d^	Estimate based on personal communication VicHealth
On-cost loading on national and state coordinator salaries	50%, 60%, 70%^c^	Triangular^d^	Estimate
On-cost loading on teachers, local government officer salaries	20%, 30%, 40%^c^	Triangular^d^	Estimate
School liaison officer time/coordinator time (hours per week)	1, 4^e^	Uniform^f^	Estimates based on personal communication VicHealth
Volunteer time (minutes per journey/day)	12, 40, 80^c^	Triangular^d^	[15]
Manual cost	$40 ± 20%^c^	Triangular^d^	Estimate
Kit bags - cost	$50 ± 20%^c^	Triangular^d^	Estimate
Special events, theme days etc.	$100, $300, $500^c^	Triangular^d^	Estimate

^a ^These values inserted separately for height and weight of boys and girls in 5-9 and 10-11 year age groups.

^b ^In a normal distribution, values are distributed in a normal bell-shaped around the mean. The distribution is based on the mean and the standard error.

^c ^Values are minimum, most likely and maximum.

^d ^In a triangular distribution, the greatest probability of being chosen is the value representing the top of the triangle (i.e. the most likely value), while the probability of other values being chosen tapers off towards the extremes of the base of the triangle (i.e. the minimum and maximum values).

^e ^Values are minimum and maximum.

^f ^In a uniform distribution, every value in the specified range has an equal probability of being chosen in each iteration of the simulation.

WSB Walking School Bus; SE Standard Error; METS metabolic equivalent units

Source: ACE-Obesity project

### Sensitivity analysis

Sensitivity testing was used to explore the impact on the ICERs of measures designed to trim costs and enhance recruitment. The following scenarios were modelled as univariate sensitivity tests: attribution of 50% of costs to non-obesity related objectives (such as reducing traffic congestion); annuitisation of fixed costs; improvements in capacity utilisation and recruitment (by increasing the number of children per WSB, WSBs per school and schools per local government, and by increasing the proportions of local governments involved and of participants new to active transport). The impact of combining these measures was tested under both an "optimistic" and a "very optimistic" scenario.

## Results

### Incremental cost-effectiveness

The incremental effect of the WSB intervention was a reduction of 0.03 (95%UI 0.01; 0.11) BMI units per child taking up walking to school (Table 3). This is about double that in Table 1 primarily due to the uncertainty range used around the number of days walked per week.

**Table 3:** Cost-effectiveness results ($AUD)

Total BMI units saved Median BMI reduction per child (new to active transport and WSB participation)	270 (40; 1,300) 0.03 (0.01; 0.11) -- boys and girls were the same
Total DALYs saved DALYs saved per person (new to active transport and WSB participation)	30 (7; 104) (excluding taggers) Males 0.003 (0.00034; 0.0086); Females 0.0018 (0.00072; 0.0079)
Total intervention cost	$22.8 M ($16.6 M; $30.9 M)
Total intervention cost by sector	
'C1': health sector	-
'C2': client/family	$2 M (8.7% of total cost) [All time costs]
'C3': other sectors	$21 M (91.3% of total cost) [Key sectors: Education]
Gross cost per BMI unit saved	$87,000 ($18,000; $490,000)
Gross cost per DALY saved	$0.77 M ($0.24 M; $3.2 M)
Total cost-offsets	$0.24 M ($0.05 M; $0.86 M)
Net cost per DALY saved (with cost-offsets)	$0.76 M ($0.23 M; $3.32 M)

* Values are medians; figures in brackets show the 95% uncertainty interval

$AUD Australian dollars; BMI body mass index; WSB Walking School Bus; DALY disability-adjusted life year; M million

Source: ACE-Obesity project

The incremental costs associated with the intervention were $22.8 M, which translated to an average cost of around $2,900 per year to get one child walking to and from school one day per week (=$22.8 M/7,840). The majority of costs related to central coordination (at national and state level) and to the recruitment of local governments and schools. The local government project officer time was by far the largest single component of costs ($14.4 M or 63%). Fixed costs constituted $19.5 M or 86% of total costs. Volunteers incurred $1.8 M (7.9%), all of which constituted time costs.

**Figure 3:**
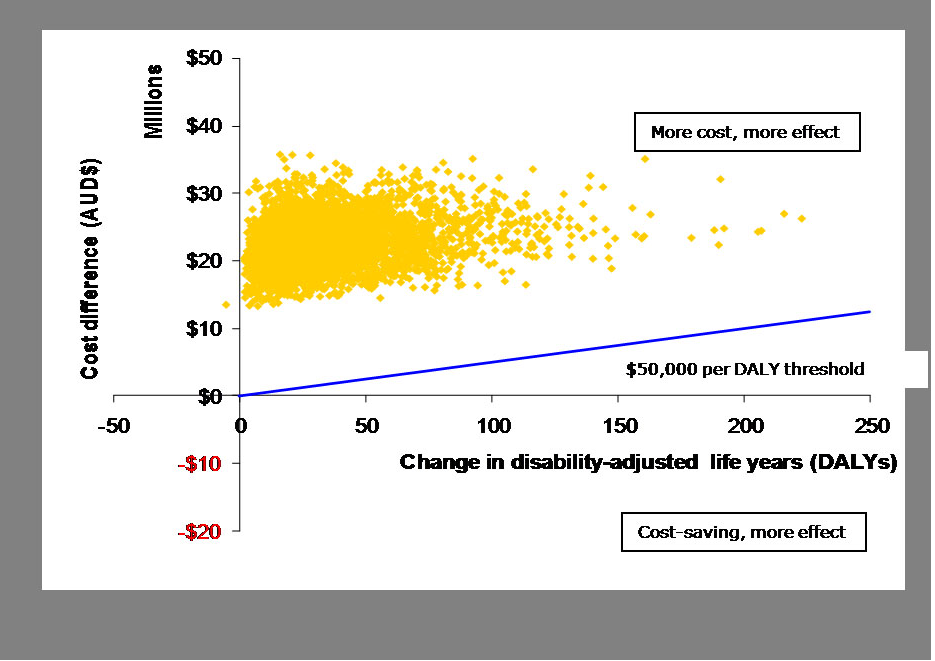
**Cost-effectiveness plane -- Net cost per disability-adjusted life year (DALY) saved (with cost-offsets)**.

The intervention produced modest cost-offsets arising from future reductions in obesity-related diseases (Table 3). The resultant net ICER of $0.76 M per DALY saved far exceeded the commonly used threshold value of cost-effectiveness in Australia of $50,000 per DALY. All of the iterations fell within Quadrant 2 of the cost-effectiveness plane, in that additional costs were incurred in achieving the health gain arising from the intervention (Figure 3). The probability of the ICERs being less than $50,000 was zero. The ICERs were unacceptably high on base run assumptions (both with and without volunteer costs), and the uncertainty intervals very wide. The main contributor to uncertainty in the ICER (cost per BMI) was the number of days of active transport per week (r = 0.56), which ranged from 0.5 (one way, one day) to 5 (both ways, 5 days) in our modelling, with 1 being the most likely value (Table 2).

### Sensitivity tests results

Cost-cutting measures including attribution of a portion of the total intervention costs to non-obesity related objectives and the annuitisation of fixed costs alone were insufficient to make the intervention cost-effective (Scenarios 2-4, Table 4). When the improved capacity utilisation measures (Scenarios 5-8) were added to the cost cutting measures (Scenario 11), the intervention was cost-effective, but only under the 'very optimistic" scenario. The inclusion of an increase in the proportion of children receiving the benefit meant the intervention approached cost-effectiveness under the "optimistic" scenario (Scenario 13).

**Table 4:** Sensitivity testing ($AUD)

**Scenario**	**Net cost per DALY saved** **(+ cost-offsets)**	
**1. Base case**	$0.76 M ($0.23 M; $3.32 M)	
**Measures to reduce costs**		
2. Attribute 50% of costs to non-obesity related objectives	$0.37 M ($0.1 M; $1.5 M)	
3. Annuitise fixed costs including volunteer training, route design and assessment, kit bags and manuals	$0.73 M ($0.22 M; $3.17 M)	
4. Combine cost-cutting measures (Scenarios 2 & 3)	$0.36 M ($0.11 M; $1.5 M)	

**Measures to improve capacity utilisation and participation **	**Optimistic**	**Very optimistic**
(using new cost base from Scenario 4)		
5. Increase no. of children per WSB from 7	to 10 $0.26 M ($82,000; $1.0 M)	to 14 $0.18 M ($58,000; $0.77 M)
6. Increase no. WSBs per school from an average of 1.6	to 3.0 $0.23 M ($71,000; $1.0 M)	to 6.0 $0.13 M ($38,000; $0.53 M)
7. Increase the number of schools involved per local government from 4	to 6 $0.27 M ($83,000; $1.1 M)	to 8 $0.22 M ($70,000; $0.9 M)
8. Increase % of non-Victorian local governments involved from 50%	to 65% $0.37 M ($0.11 M; $1.6 M)	to 75% $0.36 M ($0.1 M; $1.6 M)
9. Combine scenarios 5 and 6	$0.16 M ($57,000; $0.59 M)	$61,000($19,000; $0.25 M)
10. Combine scenarios 5, 6 and 7	$0.12 M ($45,000; $0.43 M)	$38,300 ($11,000; $0.154 M)
11. Combine scenarios 5, 6, 7 and 8	$0.118 M ($43,000; $0.4 M)	$38,000 ($11,000; $0.149 M)

**Increase participants receiving benefit**		
(using new cost base from Scenario 4)		
12. Increase % participants new to active transport from 50%	to 65% $0.28 M ($87,000; $1.1 M)	to 80% $0.22 M ($68,000; $0.89 M)
13. Combine scenarios 11 and 12	$86,000 ($35,000; $0.29 M)	$20,000 ($4,400; $83,000)

$AUD Australian dollars; DALY disability-adjusted life year; M million

Source: ACE-Obesity project

### Second stage filter analysis

A consideration of 2nd stage filters for the intervention is summarised in Table 5. The key decision points relate to the weak evidence of effectiveness for the intervention, and some significant concerns around its feasibility and sustainability. The program potentially offers some significant wider positive side-effects.

**Table 5:** Second stage filter analysis

**Level of evidence**	**Equity**	**Acceptability**	**Feasibility**	**Sustainability**	**Side-effects**
May be effective: No Level I or II evidence Modelling based on Level IV evidence Further effectiveness data sought but does not appear to support intervention's effectiveness.	Moderate equity concerns: Less access for children in rural and remote areas	Less acceptable to older children in primary schools Requires a big time commitment from the school community, which may not be acceptable to all schools	Likely issues: Variations in service delivery model between states make national implementation complex and question of appropriate auspicing body difficult. Substantial fixed costs for set-up and ongoing maintenance	Likely issues: Program requires ongoing funding & support, which may impact on sustainability.	***Positive:*** Less traffic, pollution, safer around schools. Facilitates social networks. Increases awareness of local neighbourhoods Enhances pedestrian skills. May be positive impacts on family travel behaviour ***Negative:*** Potential to decrease the number of parents walking

**Decision points** **: Weak evidence of effectiveness**	**Moderate issue**	**Not a major issue**	**Some significant concerns**	**Issue which needs to be addressed**	**Significant wider positive benefits**

**Policy considerations:** The WSB intervention is not cost-effective in terms of its effect on obesity in children under current uptake results. Action to improve uptake is worthy of consideration. Further, the intervention was not designed as an obesity prevention initiative, but as a program to produce change in the travel behaviour of students and to promote a safer traffic environment around schools. Lack of data on the incremental change in the numbers taking up active transport as a consequence of the intervention is a key limitation.					

Source: ACE-Obesity project

## Discussion

The WSB intervention was not effective or cost-effective in terms of its effect on obesity in children under the current assumptions. The ICERS, even with cost-offsets included, were very large. The reasons for the intervention's poor performance in terms of its cost-effectiveness are several. Firstly, it was modelled on the basis of the relatively low recruitment and uptake of the program in Victoria as at 2004.

It could be reasonably argued that the program has not yet reached steady state. There appears to be considerable unutilised capacity within the current delivery model, and consequent scope for expansion of the program within the current infrastructure arrangements. The number of participating local governments and schools, the number of WSBs per school, and children per bus, could all potentially be increased without expansion of the infrastructure capacity at either the national, state, local government or school level. Sensitivity testing showed that improvements on a number of fronts are required to bring the intervention into the realm of cost-effectiveness as an obesity reduction measure. However, whilst cost-effectiveness could be improved by a more comprehensive coverage and uptake of the program, the number of children required to participate at these levels may not be realistic in the Australian context.

The WSB was not designed as an obesity prevention initiative, despite often being marketed as such . The program was intended to produce change in the active transport behaviour of students. It has a number of objectives, all of which may result in potential positive side-effects (such as a safer traffic environment around schools, reduced congestion, accidents and pollution), provided that the intervention is shown to be effective in increasing the number of children new to active transport. Whilst these other benefits were documented in the second stage filter analysis, they were not incorporated into the technical results. It could be argued that the costs associated with the intervention should not have been fully attributed to the BMI outcomes, but be apportioned across a range of program objectives as was done under Scenario 2 of the sensitivity analysis. Given our adoption of a societal perspective, the choice was to capture all benefits and all costs, or alternatively, to capture only benefits and costs relevant to our objective. The latter path was chosen as data were lacking on broader benefits enabling their quantification. Whilst this evaluation concluded that the WSB was not a cost-effective intervention for obesity, its merits and potential benefits in terms of a range of other outcomes are acknowledged [17]. However, given the high cost of the intervention in terms of each child new to walking to school ($2,900) under the base case assumptions, the other benefits would need to be substantial.

Incorporation of the multiple benefits of the program within the technical analysis was neither appropriate nor possible within the context of the ACE-Obesity study. The ACE methodology is premised on the application of consistent evaluation methods to all of the interventions, and the use of the same outcome measures (in this case, BMI and DALYs saved). Cost-effectiveness and cost-utility analyses were employed to facilitate comparison with the other interventions where the same outcomes were measured. The other benefits are, firstly, not relevant to the focus of the current study, and secondly, no measurement has been undertaken within the WSB program to show that such perceived benefits have actually been realised by the program. An economic evaluation of the WSB program as a stand-alone intervention taking into account all its potential benefits would necessitate new empirical data collection around a range of outcomes, and the potential use of different evaluation methodologies (such as cost-benefit analysis where a monetary value is attached to both costs and benefits).

It may be possible to justify programs which have low direct effects such as WSB and changing school canteens [18] as 'lighthouse' interventions - meaning that they are visible demonstrations of appropriate actions that 'show the way' for broader actions and eventually changes in norms. Indeed, it is difficult to envisage a broad community effort to promote active transport without a program on school travel and healthy eating for children without school canteen changes. The counter argument to this 'lighthouse' approach is that it fosters a disregard for the importance of applying guidelines of efficiency and effectiveness in decision-making. Acceptance of the 'lighthouse' argument would need to be context specific with strong safeguards built-in around ongoing monitoring and evaluation to achieve 'value-for-money'. Our analysis suggests that more cost-effective programs to promote active transport are required and need to be evaluated. This could be achieved through improvements to the WSB or by different programs given the high cost of the WSB program as it is now.

Lack of data on the incremental change in the numbers taking up active transport as a consequence of the intervention was a key limitation of the evaluation. The limited evidence of effectiveness made the strength of evidence a key decision point. Data used in the modelling of this intervention was drawn from a one week snapshot of the VicHealth program [15]. Only 26 of the 33 participating local governments submitted data, and program activity levels may be under-estimated. However, there are no data showing an increase in the number of children walking due to the WSB intervention. Other important decision points relate to the program's feasibility and sustainability without ongoing funding (Table 5). The information gaps identified highlight the need for better evaluations of public health interventions. Whilst modelling as a tool allowed decisions to be based on the best available data, the results for this intervention are not as robust as they would be if a stronger study designs (such as RCTs) had been used and actual outcomes (i.e. BMI) were measured.

The intervention offers some potential wider positive benefits which were not taken into account. For example, the inclusion of taggers (generally older children, who were not formally enrolled in the WSB program, but "tagged" behind the buses), would increase the number of children participating in the intervention on a national basis from 15,680 to around 19,300. For the taggers, a mean reduction of 0.019 BMI units for boys and 0.021 for girls was calculated and applied. This would enhance the intervention's cost-effectiveness as it would increase the total loss in BMI, without any additional associated costs. However, it should be noted there were no data to verify that these children were new to active transport following the introduction of the WSB program. Further work is required to establish whether there is an increase in the number of children walking and in the time spent in walking and whether the other possible benefits of the program are realised.

Furthermore, this still may constitute a conservative estimate of the benefit, as the intervention involves some whole-of-school activities which may have positive spin-offs to both the wider student population as well as to parents and the wider community. Wider reach and lower costs would improve the WSB credentials as a priority for funding. The central importance of school travel in the quest to increase active transport suggests that improvements in the WSB or its variants need to be developed and fully evaluated.

## Conclusion

Whilst, under current modelling assumptions, the WSB program is not an effective or cost-effective measure to reduce childhood obesity, its economic credentials would be improved by more comprehensive implementation within current infrastructure arrangements. The attribution of some costs to non-obesity objectives (reduced traffic congestion and air pollution etc.) is justified to emphasise the other possible benefits. The importance of active transport to school suggests that improvements in WSB or its variants need to be developed and fully evaluated.

## Abbreviations

ACE: Obesity Assessing Cost-Effectiveness of Obesity; BMI: Body Mass Index; DALY: Disability-adjusted life year; EFT: equivalent full-time; ICER: Incremental Cost Effectiveness Ratio; kg: kilograms; kJ: kilojoules; M: Million; MET: Metabolic Equivalent Unit; SE: standard error; USA: United States of America; WSB: Walking School Bus; $AUD: Australian dollars.

## Competing interests

The authors declare that they have no competing interests.

## Authors' contributions

MM conducted the modelling and analysis of the intervention and assumed primary responsibility for writing of the journal article. MH project managed the ACE-Obesity project and made substantive input into the drafting of the paper. LG was responsible for the literature review and data collection underpinning the modelling. BS provided expertise in the modelling from a change in physical activity to a change in BMI, and also provided valuable comment on the paper. RC provided technical expertise around the economic evaluation protocol and modelling, and provided comment on the paper. All authors read and approved the final manuscript.

## Supplementary Material

Additional file 1 **Unit costs, data sources and assumptions**. Unit costs are provided for all resources used in the delivery of the intervention. Data sources are specified, as well as any assumptions employed. $AUD Australian dollars; EFT equivalent full-time; WSB Walking School Bus Click here for file
