# Supplementary material for: Cost-effectiveness of active transport for primary school children - Walking School Bus program
Source: Int J Behav Nutr Phys Act. 2009 Sep 14;6:63. doi: 10.1186/1479-5868-6-63 (PMC2758827; doi:10.1186/1479-5868-6-63)
Supplement: Additional file 1 — Unit costs, data sources and assumptions. Unit costs are provided for all resources used in the delivery of the intervention. Data sources are specified, as well as any assumptions employed. $AUD Australian dollars; EFT equivalent full-time; WSB Walking School Bus [file 1479-5868-6-63-S1.doc]

**Additional file 1. Unit costs, data sources and assumptions**

| **Element costed** | **Unit cost**  **2001**  **$AUD** | **Source** | **Assumptions** |
| --- | --- | --- | --- |
| **Central coordination and recruitment of local governments and schools** | | | |
| Program coordination (per officer per annum) | $84,152.00 | Middle of Australian Public Service Level 6 salary range | Includes 60% on-costs to cover salary on-costs, office overheads, consumables, administrative support and routine monitoring, support and evaluation. Assume 9 EFT (one Commonwealth and one per state). |
| Local government preparation of submission (per day) | $262.98 | Middle of Australian Public Service Level 6 [www.apsc.gov.au](http://www.apsc.gov.au/) | Includes 30% salary on-costs only, and no overheads as assumed to be an existing position. Allows 4 weeks of local government officer time (annuitised over 5 years) to prepare submission to participate in WSB. |
| Panel assessment of submissions (per day) | $262.98 | Middle of Australian Public Service Level 6 [www.apsc.gov.au](http://www.apsc.gov.au/) | Includes 30% salary on-costs only. Submissions externally assessed by panel of government representatives. Allow 40 minutes per submission (annuitised over 5 years). |
| Local government project management and liaison (per day) | $262.98 | Middle of Australian Public Service Level 6 [www.apsc.gov.au](http://www.apsc.gov.au/) | Includes 30% salary on-costs only. Successful Councils appoint a 0.6EFT to provide day-to-day management and liaison with schools. |
| School WSB liaison person (per day) | $236.62 | Subdivision A-3 of Accomplished Classroom Teacher range. Victorian Department of Education and Training, Salaries for Teacher Class Structure, August 2004 | Includes 30% salary on-costs only, and no overheads as assumed to be an existing position. In each school, one teacher allowed one hour per week (40 weeks) to be WSB liaison person. |
| **Recruitment, registration and training of volunteers** | | | |
| Volunteer attendance at recruitment meeting (per hour) | $15.47 | Australian Bureau of Statistics [1] | Assume 15 persons attended for 2 hours. Opportunity cost principle used in valuing volunteer time costs. Assume 90% volunteers not engaged in paid employment, and therefore sacrifice leisure time (equal to 25% Australian average weekly earnings rate) [1,2] |
| Volunteer insurance | Nil |  | Local governments have existing insurance policy. Premium unaffected by registration of new volunteers. |
| Conduct of volunteer training by Vic Roads officers (per day) | $262.98 | Middle of Australian Public Service Level 6 [www.apsc.gov.au](http://www.apsc.gov.au/) | Includes 30% salary on-costs only, and no overheads as assumed to be an existing position. Allow 3 hours of officer time per two hour training session. Training provided to individual schools, and repeated once annually for 50% schools as new volunteers enter program. |
| Volunteer attendance at training | $15.47 | Australian Bureau of Statistics^20^ | Allow 2.5 hours per volunteer (includes 15 minutes travel each way). Assume 90% volunteers not engaged in paid employment, and therefore sacrifice leisure time (equal to 25% Australian average weekly earnings rate) [1,2] |
| Training venue hire | $110.00 | Estimate | Current (2004) figure of $120 |
| Catering at training session (per head) | $27.63 | Estimate | Based on current(2004) estimate of $30 |
| Police checks of volunteers | $11.33 | Victoria Police website  [www.police.vic.gov.au](http://www.police.vic.gov.au/) | One check per volunteer. Price currently paid by VicHealth ($12.30 in 2004) |
| **Local planning phase** |  |  |  |
| Identification of WSB routes | $15.47 | Australian Bureau of Statistics^20^ | Assume one hour for 2 volunteers per route. No additional time allowed for School WSB liaison person or Council project officer as part of their yearly time allocation. |
| Route assessment:  (i) Local government coordinator, community safety officer and traffic engineer  (ii) Volunteers | $262.98  $15.47 | Middle of Australian Public Service Level 6 [www.apsc.gov.au](http://www.apsc.gov.au/)  Australian Bureau of Statistics [1] | Route assessment to identify any infrastructure and safety issues. Includes 30% salary on-costs only. Allow 2 hours of Local government staff time  Allow one hour per two volunteers per route. |
| Photocopying promotional material (per page) | $0.05 | Commercial rate | Promotional material distributed to parents of 20 potential participants per route |
| **Routine operation of WSB** | |  |  |
| Volunteer operation of WSB | $15.47 | Australian Bureau of Statistics [1] | Allow for 40 minutes per volunteer per round trip^16^. Gender specific average ordinary time earning adjusted to labour force statistics and population gender ratio^18^. Assumes 50% volunteers were walking to school previously (no cost assigned), 40% non-working volunteers (assigned leisure time rates = 25% unit costs, 10% working volunteers (100% unit cost) [2] |
| Kit bags | $50.00 | Estimate | Based on advice from VicHealth. Contain essential items, such as a clipboard with attendance sheets, pens, a whistle, and fluorescent vests or arm bands. |
| Curriculum manuals | $40.00 | Estimate | Based on advice from VicHealth |
| Blanket amount to cover special events, theme days etc. | $300 | Estimate | For promoting the program and engaging the school community. |

[1] Australian Bureau of Statistics: Average weekly earnings, Australia Cat. 6302.0, 2001. Melbourne: Australian Bureau of Statistics; 2001.

[2] Jacobs P, Fassbender K: **The measurement of indirect costs in the health economics evaluation literature. A review.** *Int J Tech Assess Health Care* 1998,**14**:799-808.
